# Mesoporous Silica Nanoparticles: Properties and Strategies for Enhancing Clinical Effect

**DOI:** 10.3390/pharmaceutics13040570

**Published:** 2021-04-17

**Authors:** Alex N. Frickenstein, Jordan M. Hagood, Collin N. Britten, Brandon S. Abbott, Molly W. McNally, Catherine A. Vopat, Eian G. Patterson, William M. MacCuaig, Ajay Jain, Keisha B. Walters, Lacey R. McNally

**Affiliations:** 1 Stephenson School of Biomedical Engineering, University of Oklahoma, Norman, OK 73019, USA; africk256@ou.edu (A.N.F.); Catherine.A.Vopat-1@ou.edu (C.A.V.); wmaccuaig@ou.edu (W.M.M.); 2 Stephenson Cancer Center, University of Oklahoma, Oklahoma City, OK 73104, USA; Jordan-Hagood@ouhsc.edu (J.M.H.); molly-mcnally@ouhsc.edu (M.W.M.); 3 School of Chemical, Biological, and Materials Engineering, University of Oklahoma, Norman, OK 73019, USA; collin.britten@ou.edu (C.N.B.); brandonabbott@ou.edu (B.S.A.); keisha.walters@ou.edu (K.B.W.); 4 Department of Biology, University of Oklahoma, Norman, OK 73019, USA; eian_pat@ou.edu; 5 Department of Surgery, University of Oklahoma, Oklahoma City, OK 73104, USA; Ajay-Jain@ouhsc.edu

**Keywords:** mesoporous silica, theranostics, theragnostics, nanomedicine, cancer, active targeting, toxicity, controlled release

## Abstract

Due to the theragnostic potential of mesoporous silica nanoparticles (MSNs), these were extensively investigated as a novel approach to improve clinical outcomes. Boasting an impressive array of formulations and modifications, MSNs demonstrate significant in vivo efficacy when used to identify or treat myriad malignant diseases in preclinical models. As MSNs continue transitioning into clinical trials, a thorough understanding of the characteristics of effective MSNs is necessary. This review highlights recent discoveries and advances in MSN understanding and technology. Specific focus is given to cancer theragnostic approaches using MSNs. Characteristics of MSNs such as size, shape, and surface properties are discussed in relation to effective nanomedicine practice and projected clinical efficacy. Additionally, tumor-targeting options used with MSNs are presented with extensive discussion on active-targeting molecules. Methods for decreasing MSN toxicity, improving site-specific delivery, and controlling release of loaded molecules are further explained. Challenges facing the field and translation to clinical environments are presented alongside potential avenues for continuing investigations.

## 1. Introduction

Small molecule approaches to diagnostic and therapeutic procedures suffer from multiple shortcomings, such as off-target toxicity and rapid clearance from the body. Boasting the ability to simultaneously diagnose and treat patients, theragnostic (a.k.a. theranostic) nanoparticles (NPs) emerged as a rapidly developing technology for overcoming these obstacles and enhancing clinical outcomes. In particular, mesoporous silica nanoparticles (MSNs) demonstrate a significant potential [1] to become a standard part of the therapeutic armamentarium for various diseases. MSNs possess sufficient biocompatibility, particularly compared to other inorganic NPs, alongside highly structured and stable porous networks, into which drug or dye molecules can be loaded in large quantities. Additionally, the size, shape, and pore properties of MSNs can be highly controlled during synthesis reactions [2], providing multiple NP formulations from the same composite elements.

Pairing with appropriate gatekeeper molecules can trap encapsulated molecules within the MSN pores, allowing for efficacious cargo release. In addition, the gatekeeper provides surface functionalization to prevent toxicity. These gatekeeper mechanisms protect the nontarget tissues—in the host environment—from loaded molecules, while simultaneously protecting the loaded molecules from the environment. This two-way shielding provided by MSNs allows for in vivo delivery of small molecules that, alone, would be clinically ineffective as a result of their high hydrophobicity or toxicity [3].

The multifunctional nature of MSNs largely stems from the numerous surface modifications they can undergo. As mentioned, many different gatekeeper molecules can be employed to the encapsulated molecules loaded into MSN pores. These gatekeeper molecules can be chosen or designed to respond to disease-specific stimuli, thus, controlling the release of loaded molecules and limiting off-target delivery [4]. The organic nature of gatekeeper molecules also serves to limit immunogenic responses from the host that might otherwise occur. If unmodified MSNs were administered with or without gatekeepers, MSN surfaces could be effectively modified to specifically target them to selected tissues or sites of disease. This feature differentiates MSNs from NP formulations that might rely solely on passive targeting mechanisms to reach their target destination. The number of possible active targeting molecules is vast and ever growing, further promoting the application of MSNs in identifying and treating many different diseases.

**Figure 1:**
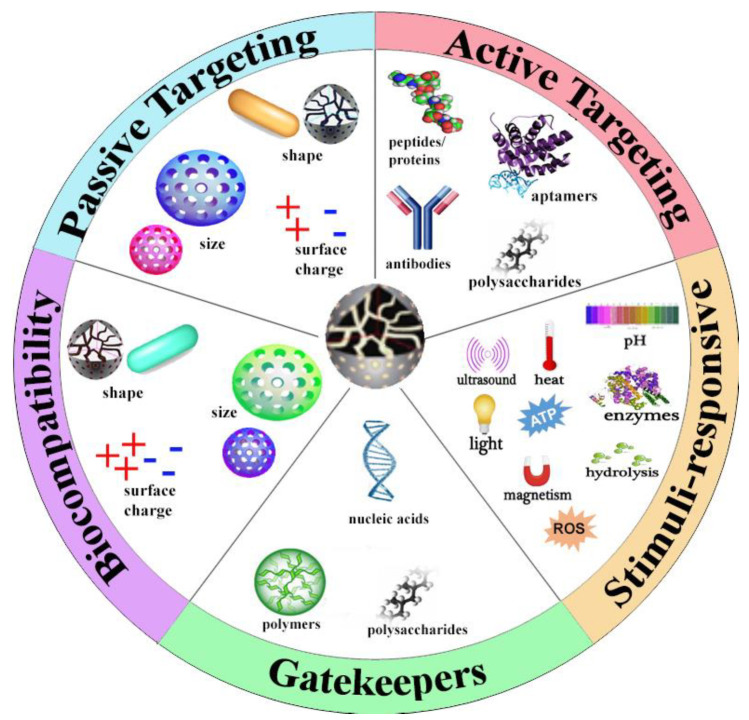
Characteristics of MSNs that drive theragnostic effect.

Compared to the number of preclinical studies, very few silica-based nanomedicine approaches were FDA approved for clinical trials [5,6]. Continued investigation of MSN formulations is contingent upon understanding the myriad factors influencing MSN clinical efficacy. As MSN technology advances, characteristics of MSNs and their correlating structural modifications to function, specificity, and toxicity needs to be well understood. This review highlights recent advances with MSNs relative to cancer theragnostics. MSN properties (see Figure 1) that promote clinical efficacy are detailed with a particular focus on surface properties and toxicity. Targeting strategies employed by MSNs are similarly discussed, highlighting the advantages and options for active targeting methods. Theragnostic actions of MSNs are discussed as well, in addition to the many clinical applications and strategies to reduce the potential toxicities from the MSNs. Finally, future directions for the field are briefly analyzed, relative to enhancing clinical translation.

## 2. Characteristics of MSNs

**Figure 2:**
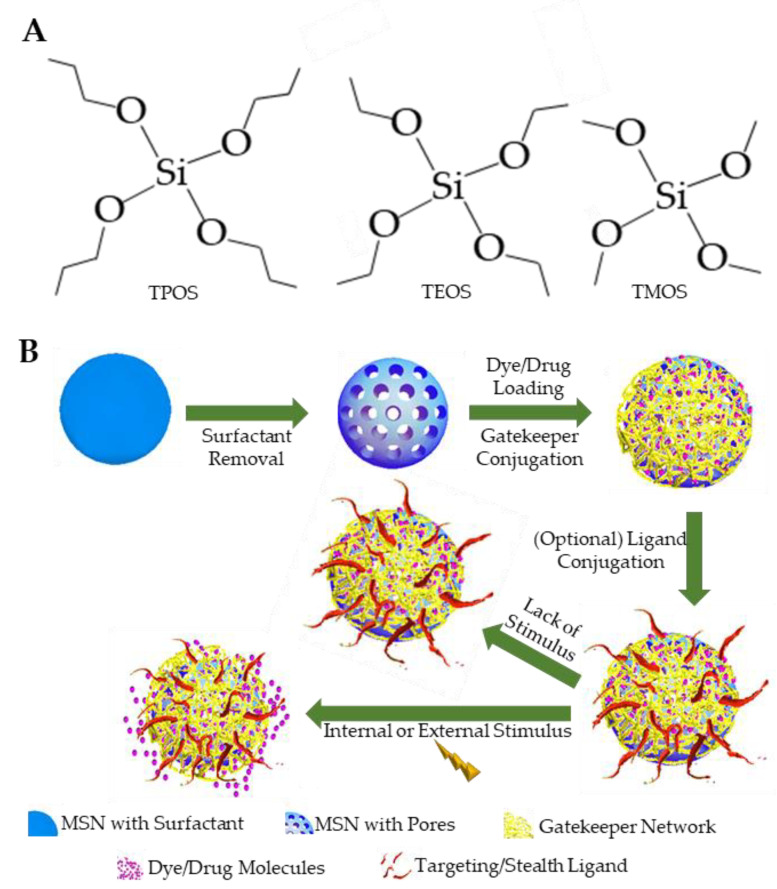
MSN precursors and theragnostic action. (**A**) The common orthosilicate precursors used in MSN synthesis reactions. (**B**) MSN synthesis, loading, and controlled release. Note that the surface of the MSN is coated with a biocompatible gatekeeper. The biocompatible gatekeeper has a dual role in that it allows retention or release of dye/drug molecules and facilitates biocompatibility of the nanoparticle to reduce toxicity. A stimulus can result in swelling or destruction of a stimuli-responsive gatekeeper. A dye/drug can release based upon stimuli-responsive changes in the gatekeeper, regardless of the presence/absence of an active targeting molecule by diffusion.

While many reaction pathways might be used [7,8], MSNs are primarily synthesized through sol–gel reactions [9,10,11,12]. Surfactant molecules, such as hexadecyltrimethylammonium bromide (CTAB), are dissolved in ultrapure water, prior to addition of cosolvents and tetralkoxy silicate precursor molecules. The most commonly used silicate precursor used in MSN synthesis is tetraethyl orthosilicate (TEOS). Tetrapropyl orthosilicate (TPOS) and tetramethyl orthosilicate (TMOS) are alternative options used in some studies, though TEOS is favored, given the apparent ease to control TEOS reaction output [7,13]. Selection of surfactant molecule, cosolvents (typically alcohols or strong bases), reaction temperature, and magnetic stir speed, all determine the size, shape, and porosity of the synthesized MSN product. Investigations into the relation between silicate precursor and particle size are ongoing. Following synthesis of MSN core particles, template surfactant molecules, such as the commonly used CTAB, must be removed to maximize effective pore volmers. Additionally, surfactant molecules can be toxic in vivo, as is the case with CTAB [14,15,16,17], requiring their removal from MSN pores, prior to clinical applications. Several methods for surfactant removal might be applied, including washing with water and ethanol, dialysis [18], or calcination [19]. Surfactant removal might be followed by additional steps that modify core MSN functional groups, conjugate a gatekeeper system to the MSN surface, or conjugate active targeting ligands to the MSN nanovehicle. Figure 2 provides an overview of the functionalization and theragnostic action of MSNs.

Strategically tailoring MSN surface-oriented functional groups provides avenues to better control interfacial interactions, while offering additional functionality like drug loading and insertion of targeting domains. Both chemical treatment and polymer grafting strategies are widely used approaches for chemically or morphologically modifying MSN surfaces [20,21]. Self-assembled monolayers (SAMs), such as silanes on hydroxylated surfaces, offer one facile and flexible approach at introducing a wide array of functional groups to MSN surfaces. Comprising a head group, a hydrocarbon-based backbone, and a functional end group, silane-based SAMs spontaneously adsorb onto the hydroxylated surface of MSNs. Afterwards, SAMs self-organize to form larger structured domains (i.e., ca. 1–3 nm organic thin films) and become well-ordered through van der Waals interactions. The result provides close-packed terminal groups that can be used as initiation sites for grafting polymeric material [22]. Through the initiation of the surface-oriented SAM functional groups, polymers exhibiting a plethora of confirmations and arrangements can be surface-confined (i.e., grafted). Tailoring the grafting and polymerization approach allows for the synthesis of reproducible, well-defined, polymeric brushes with tunable macromolecular properties that enhance the MSN theragnostic potential.

Beyond the impact of polymeric, gatekeeper, or active targeting molecules, innate properties of the core MSN serve as key predictors of physiological fate. Prior to administration, complete characterization of MSNs is critical to approximating MSN behavior and correlating the theragnostic effect. Some of the common methods for verifying MSN properties by analyzing synthesized particles are dynamic light scattering (DLS) [23] and transmission electron microscopy (TEM); and by analyzing the specific surface area calculations through the Brunauer–Emmett–Teller (BET) methods [24]. Understanding how the tunable traits of MSNs influence potential theragnostic action is critical to optimizing clinical efficacy.

### 2.1. Size

Several organ systems within the body possess structural or functional filtration abilities. For example, the glomerular layers of the kidney are responsible for filtering foreign and waste products that are smaller than approximately 5 nm [25,26]. Simultaneously, the highly vascularized liver and spleen uptake particulates of 100 nm from the bloodstream for removal via the reticuloendothelial system (RES), through Kupffer cells or splenic macrophages, respectively [27,28,29,30,31]. Given these risks of RES clearance and excessive renal filtration, it is important to synthesize particles that are of appropriate size for maximum retention time and clinical efficacy. NPs within the size range of 20–80 nm appear to be most favorable for avoiding the various physiological “traps” in the body, while promoting target site accumulation [32,33,34]. Size-based studies of MSNs demonstrate the increased ability of MSNs within this size range to reach target sites relative to larger MSNs [35,36,37]. Based on these size restrictions, MSNs synthesized from TEOS might be less effective for in vivo application, depending on the synthesis method employed. MSNs comprised of TMOS suffer the disadvantage of an increased tendency to aggregate after synthesis [7,10], but this issue is largely overshadowed by their smaller size of ~30 nm, prior to modification [38].

While control of the MSN size is primarily accomplished prior to treatment, it is critical to consider the fact that significant changes in the nanovehicle size might occur from physiological reactions in vivo. Following intravenous administration, MSNs are exposed to numerous proteins in the blood. Under certain conditions, these proteins can irreversibly bind the MSN surface through a process termed as opsonization [39,40]. The resulting protein corona, with the NP in the center, has an increased effective size relative to the original MSN. This can result in increased splenic or liver sequestration and clearance. Opsonization might decrease the likelihood that the MSNs would be immediately recognized as foreign material and subsequently trigger an immunological response [41]. This could result in increased MSN circulation time, although the interaction with target cells is hampered by the large proteins bound to the nanovehicle surface. The magnitude and variety of protein content in the corona was found to vary significantly, based on MSN size and surface functionalization [42,43,44]. Additionally, endogenous biomolecules exposed to or existing as part of the protein corona might undergo structural and functional alterations [45]. Such alterations might activate undesired signaling pathways or immune cells, ultimately leading to immunogenic or toxic side effects [46,47,48]. Protection against opsonization could be achieved by conjugating organic polymers or biomolecules (such as gatekeepers or targeting ligands) to the surfaces of MSNs [49,50,51].

### 2.2. Shape and Porosity

**Figure 3:**
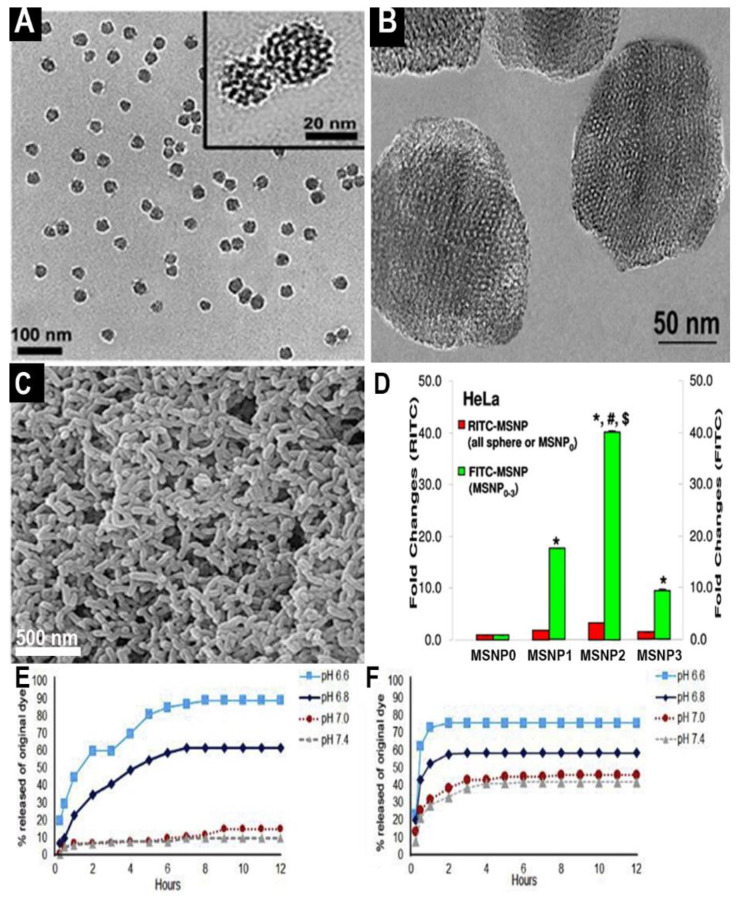
Shape and porosity differences between MSNs influence outcomes. (**A**) Wormhole pore MSNs with a particle diameter of 25 nm and a pore diameter of 1.3 nm, reprinted with permission from [38], Elsevier, 2018. (**B**) Honeycomb pore MSNs with a particle diameter of 130 nm. (**C**) Rod-shaped MSNs synthesized from TEOS, reprinted with permission from [60], Elsevier, 2020. (**D**) MSN rods of AR ≈ 2.1–2.5 (MSNP2) exhibited high uptake in HeLa cells, according to the RITC and FITC analysis, as compared to spheres (MSNP0) and rods of smaller (MSNP1) and larger (MSNP3) AR values; * indicates *p* < 0..05 when compared with MSNP0; # indicates *p* <0.05 when compared with MSNP1; $ indicates *p* < 0.05 when compared with MSNP3. Reprinted with permission from [53], ACS Publications, 2011. (**E**) Wormhole porous MSNs with chitosan gatekeeper networks exhibit more favorable pH-specific controlled release compared to (**F**) honeycomb porous MSNs with the same gatekeeper. (**A**,**B**,**E**,**F**) reprinted with permission from [38], Elsevier, 2018.

The final shape of the MSN core plays a similarly critical role in physiological fate and in vivo MSN behavior. MSNs are most commonly shaped as spheres or rods. The final shape primarily depends on the identity and volume ratio relative to water, in the cosolvent used during the sol–gel reaction [52]. The aspect ratio (AR) of synthesized rod MSNs is similarly dependent on reaction conditions, namely temperature and magnetic stir speed. MSN shape and aspect ratio significantly affect in vivo circulation time and tissue penetration. In general, it was observed that rod shaped MSNs of sufficient AR (approximately 2.1–2.5) exhibit higher blood circulation times and tumor penetration depths, as compared to spherical MSNs or rods of different AR values (see Figure 3D) [34,53,54,55]. Rate of engulfment by target cells is also influenced by MSN shape and AR, though the results might be cell-line dependent [56]. Other MSN morphologies were also explored [57]. For example, MSNs possessing rough surfaces, demonstrated increased uptake by target cells, as compared to both solid and mesoporous silica counterparts. These unique virus-like MSNs were generated through additional synthesis steps, following the initial MSN core formation [58,59].

MSN porosity is characterized by the shape, diameter, and number of pores. Pore shape is primarily determined by the cosolvents used during synthesis. The standard honeycomb pore shape is produced when strong bases such as NaOH are used for the cosolvent [61,62]. Wormhole pores are generated when other cosolvents like triethylanolamine (TEA) are included in the synthesis reaction [38,63]. Honeycomb MSNs exhibit less restricted pore spaces and more stable colloidal suspensions than those with wormhole pores. The release of honeycomb loaded molecules, however, occurs in a less controlled fashion (resembling burst-release kinetics), as compared to the wormhole loaded agents (see Figure 3E–F) [38]. After MSN synthesis, pore size could be decreased by up to ~0.5 nm via vacuum-assisted vapor deposition of TEOS or TMOS [64,65]. Such adjustment of pore size permits finer control over the loaded molecule release rates and the functional surface area of the MSN core. While this increase in surface area might promote MSN–cell interactions, it also provides additional sites for MSNs to interact with healthy host cells, increasing the risk of toxic effects or off-target accumulation [66].

### 2.3. Surface Properties, Charge, and Toxicity

The major function of MSNs is the specific delivery of encapsulated cargo to a target location in vivo. To incorporate specificity, often a stimuli-sensitive gatekeeper is added to the surface of the MSNs. In addition to providing stimuli-responsivity for cargo release, the gatekeeper has the capability to circumvent toxicity. As the major benefit of the mesoporous nature of the MSNs allows for release of a cargo in conjunction with a particular disease, the MSN should have a gatekeeper on the surface of the MSN to allow promotion of site-specific drug/dye release. While non-coated MSNs reportedly induced proinflammatory and toxic responses, notably through accumulation in the liver and kidney [67,68], non-coated MSNs do not have cargo release specificity, hindering clinical application. Addition of gatekeeper molecules to the surface of MSNs provides the specificity for cargo release, while simultaneously ameliorating toxicity concerns.

The primary functional group present on unmodified MSNs is silanol, which has the general form ≡Si-OH [69]. The silica atom is bound to at least one oxygen atom, which could be protonated or deprotonated, depending on the environmental pH or could bind to other neighboring silica atoms [70]. The other groups bound to silica atoms are dependent on the orthosilicate molecule used to synthesize the MSN (see Figure 2A for common examples) and the reaction conditions applied. For example, the silanol group can exist in an isolated ≡Si-OH form, a germinal =Si-(OH)_2_ form, or as a series of siloxanes (e.g., ≡Si-O-Si-O-Si≡). Additionally, hydrogen bonds can form between silanols, based on group density, forming vicinal silanol groups [70,71]. At physiological pH values of ~7.4, hydrogen-containing silanol groups can become deprotonated, resulting in a net negative charge for MSN surfaces [72]. NPs with negative charges are less likely overall to interact with or be engulfed by nonphagocytic cells, thus, prolonging the NP circulation time [73,74]. This benefit of the negative charge comes at the cost of increased risk for hemolytic interactions between MSNs and red blood cells, based on the MSN negative charge [66,75,76]. The negative charges also interact unfavorably with the immune cells and functions. For example, negatively charged MSNs are shown to inhibit growth and multiplication of lymphocytes [77,78]. The negative charge might increase the rate of opsonization as well [79]. Unsurprisingly, MSN shape, size, porosity, and dose concentration influence the magnitude of these toxic effects, based on the number of negatively charged silanol groups available for interaction.

Functional group modification is assessed as a method for altering MSN cellular interactions. For example, silanol groups can be replaced with the amine functional groups for the net positive surface charge [80]. MSNs possessing amine or carboxyl surface functional groups demonstrated significantly lower immune cells cytotoxicity, as compared to the unmodified MSNs [81]. Similarly, MSNs grafted with phosphonate groups show enhanced drug delivery to tumor cells with limited undesirable cytotoxicity or opsonization [35,76], despite having a negative charge. Amine- and phosphate-modified MSNs show similarly mitigated proinflammatory responses that can be induced by the unmodified MSNs and even MSNs conjugated with poly(ethylene glycol) (PEG) [67]. Relative to protecting blood lymphocytes from damage caused by unmodified MSNs, surface modifications using vinyl and aminopropyl/vinyl functional groups exhibited a limited cytotoxic effect [82]. While the discussed options focus on MSN surface groups in relation to toxicity, surface modification was also performed to enhance MSN encapsulation and delivery of molecular cargo [83].

Maintaining non-toxicity of MSNs is crucial in the pursuit of clinical adaptation. Thus far, MSN coatings focus on alternative uses for the biocompatible materials used in applications such as wound healing [84] or topical biostimulation methods [85]. Chitosan is a naturally occurring polysaccharide with pH-responsivity and is widely regarded as a non-toxic coating for MSNs [86]. However, careful derivatization of chitosan is required to ensure no potential toxic contamination occurs, notably by surpassing injection concentration thresholds or due to reduction of the positive surface charge [87]. Hyaluronic acid (HA) is a glycosaminoglycan occurring in the extracellular matrix critical to cell growth and stability. HA was explored for topical and in vivo applications, ranging from skin rejuvenation to cancer therapy. Similar to chitosan, HA can be used in vivo without significant toxicity concerns, by adhering to the synthesis and conjugation guidelines [88]. Polydopamine and poly l-histidine are pH-sensitive polymers that could be incorporated as an MSN coat to reduce the inflammatory responses. Each of these coatings, while providing stimuli-responsivity for cargo delivery, also exhibit no cytotoxicity or indications of fibrosis [89,90]. However, it is important to note that gatekeepers must be extensively investigated prior to use as MSN coating agents; biocompatible gatekeepers might induce undesired toxicity when conjugated to MSNs. For example, poly-ethylene glycol (PEG) is often described as a non-toxic stealth coating. PEGylated nanoparticles raise toxicity concerns, notably anaphylaxis [91], based on longer blood circulation times and limited cellular uptake [92]. While such described gatekeepers show a potential for biocompatibility as MSN coatings, varying applications might provoke undesired interactions, resulting in toxicity. Future investigations would require extensive evaluations of each MSN gatekeeper to ensure elimination of toxicity concerns. In addition to offering tailorable surface chemistries and limiting toxicity, graft polymerization stabilizes MSNs through electrostatic and steric effects, allows for the synthesis of a wide array of physical structures, and provides additional material functionality [93,94]. Macromolecules are grafted to MSN surfaces through two main approaches—grafting-to and grafting-from. Grafting-to strategies attach pre-formed polymers directly to MSNs, often utilizing click chemistry to attach a functionalized end group to the MSN surface [95,96]. However, grafting-to strategies are limited in polymer density, as the steric hindrance between polymer molecules increases the distance between individual chains [97]. This can result in a higher exposure of the bare MSN surface, increased risk of MSN toxicity, and decreased polymeric functionality. Grafting-from strategies, on the other hand, rely on attaching initiator moieties to MSNs and polymerizing directly from the MSN surface. This approach allows for higher grafting densities, as well as the tailoring of more complex polymer architectures, such as partial crosslinking to further increase surface density [97].

Often, polymer synthesis strategies rely on controlled radical polymerization (CRP) techniques, which allow for the controlled design of polymer architecture, chain length, branching, functionality, tacticity, stereochemistry, and composition, among other features. CRP strategies also retain functional end groups with high fidelity [98,99,100,101,102], allowing for design of block copolymers for multiple applications. Further, these end groups might be modified for conjugation to MSN surfaces or to active targeting moieties, to reduce off-target accumulation [103]. Commonly utilized CRP techniques include atom transfer radical polymerization (ATRP), reversible addition/fragmentation chain transfer polymerization (RAFT), and nitroxide-mediated polymerization (NMP). Due to the potential toxicity of the transition metal catalyst species needed for ATRP, RAFT polymerization received considerable attention as a more clinically suited method for controlled graft polymerization [104]. Lower toxicity catalyst systems for ATRP were recently assessed, including iron-mediated and even metal-free photo-ATRP [105,106]. Ultimately, CRP strategies allowed for the design of novel MSN-composites with tunable surface properties and interfacial interactions.

Besides altering the MSN surface chemistry, conjugation of organic molecules (such as polymers) to the surface of MSNs is a common strategy for limiting unfavorable MSN–host interactions. Conjugated molecules impart addition functions to MSN nanovehicles, including gatekeeping and active targeting. Some modifications are performed to protect the NP from the host, or vice versa. The most common example of such a molecule is PEG, whose biocompatibility was demonstrated in numerous NP formulations [107]. While PEG-modified MSNs show preclinical effectiveness with limited off-target toxicity and opsonization [108,109,110,111,112], they reach disease sites through passive targeting methods. This severely limits their efficacy and therapeutic potential in humans who benefit much more from the actively targeted MSNs. Additionally, PEG molecules can inhibit MSN interactions with target cells or limit long-term clinical effectiveness, as a result of the PEG-specific antibody formation [113,114,115].

Polymers, polysaccharides, and other organic-based molecules added to the surface of MSNs can substantially alter interactions between the host milieu and the NP, resulting in reduced toxicity. These surface coatings or gatekeeper molecules must exhibit significant biocompatibility and negligible toxicity. Specific assessment of toxicity for gatekeepers was performed for select molecules, including chitosan [87,116], poly(L-histidine) [117], polydopamine [89], and hyaluronic acid [118]. In each case, the molecules demonstrated limited toxicity for both in vitro and in vivo models. Continued toxicity assessment of current gatekeeper and surface functionalization molecules that show apparent preclinical effectiveness in MSN formulations is warranted. Future studies should further consider how MSN toxicity changes after surface modification and conjugation to targeting ligands.

## 3. Molecular Encapsulation and Stimuli-Specific Release Response

Given the porous nature of MSNs and the potential toxic effects associated with the exposed functional groups, additional chemical and structural mechanisms are needed to both encapsulate molecules loaded into MSN pores and to limit unfavorable interactions between the MSN surface and the host. These “gatekeeper” systems, commonly comprised of organic polymer networks, facilitate release of loaded molecules, once the MSNs reach the target location or cell. Additionally, they serve as a surface onto which other molecules, such as active targeting ligands, could be conjugated. A selection of polymeric gatekeeper molecules that demonstrated stimuli-specific response in preclinical studies are provided in Table 1.

Controlled release of encapsulated molecules is triggered by cell- or site-specific stimuli that changes the gatekeeper system, exposing the pores of the MSN. Any loaded molecules would then diffuse out of the MSN into the target environment. Appropriate selection of gatekeeper molecule requires knowledge of any internal stimuli produced in the target cell or environment that the gatekeeper would respond to. While common stimuli are found internally, external stimuli provided by clinicians might also be used after sufficient accumulation of MSNs at the target site. Given the extensive and ever-growing number of possible gatekeeper molecules, it is more valuable to consider the methods of triggering gatekeeper stimuli-specific response. Upon identifying the stimuli that matches the target disease, an appropriate gatekeeper can be used in formulating the MSN nanovehicle.

### 3.1. Internal Stimuli

#### 3.1.1. pH

The altered metabolic behavior of malignant cells results in the synthesis of acidic byproducts that are transported to the extracellular environment [145]. This phenomenon results in a decrease in extracellular pH from 7.4 (standard physiological pH) to as low as 6.4 [146]. The lower extracellular pH in tumor tissues can be used as a strategy to facilitate targeted release of anti-cancer therapeutics agents from MSNs. Similarly, the acidic pH of endosomes and lysozymes into which MSNs are transported during endocytosis and degradation, respectively, is also used to trigger the release of encapsulated molecules [147]. The release results from the higher proton concentration, triggering a chemical shift in the selected gatekeeper molecules. For example, chitosan is a naturally occurring polymer that possesses a primary amine in its structure. At neutral pH values, the amine group is deprotonated, tightening the polymer network around the MSN. In acidic environments, the amine group is protonated, acquiring a positive charge and becoming highly hydrophilic [148]. This results in a swelling of the polymers around the pores [149], exposing pore contents to the environment and permitting diffusion of the encapsulated molecules out of the MSNs. While other functional or electrostatic groups might be used in gatekeeper molecules, the principle remains the same—acidic pH results in electrochemical changes that alter the gatekeeper network or interactions between the gatekeeper molecules and the MSN itself.

Chitosan is successfully used as a pH-sensitive gatekeeper in several MSN preclinical model studies [38,61,62,119,120,121]. Other polymers that demonstrate similar pH-sensitivity include polyvinyl pyridine [122,123], poly(L-histidine) [90,95], poly(acrylic acid) [124,125,126], gelatin [132,133], and polydopamine [112,137,138,139]. Bonds between the gatekeeper molecules and the MSNs themselves could be chosen for their response to acidic pH as well, largely resulting in dissociation of gatekeeper molecules from the MSN surface. Examples of such formulations can utilize imine bonds [150,151,152], ester bonds [150,153,154], or hydrazine bonds [155], among others [147]. In all these cases, the gatekeeper molecule is organic in nature, improving biocompatibility between the MSN nanovehicle and the host while limiting toxicity. While organic pH-gated molecules are commonly used, inorganic systems were studied as well. For example, latching mechanisms functionalized by metal ions of cobalt, nickel, or calcium show pH-specific release of molecules loaded into MSNs [156]. Zinc quantum dots were similarly studied as MSN gatekeepers, given their rapid dissolution at acidic pH values [157,158]. One system showed similar biocompatibility and pH-sensitive release, when using calcium carbonate—which dissociates into Ca^2+^ and CO_3_^2−^ ions in acidic pH [159].

#### 3.1.2. Enzymes

While pH-response can preferentially increase delivery to preferred tissues, it is fundamentally a non-specific targeting strategy that can be neutralized by changes in pH. Any change in the pH of the suspending solution would induce release of the loaded molecules. Gatekeeper motifs can be constructed to break down in the presence of more specific stimuli, such as enzymes. Malignant cells often overexpress enzymes that promote proliferative or metastatic behavior [160]. These enzymes can be found in either the extra- or intracellular environment, providing additional avenues for gatekeeper application. Selection of gatekeeper molecules that are targeted for destruction by overexpressed or lysosomal enzymes promotes controlled release.

As with pH-sensitive gatekeepers, many molecular networks stimulated by enzymes comprise polymers. Hyaluronic acid (HA) is a common example used in MSN formulations. HA is advantageous as it serves as both an active targeting molecule that binds to overexpressed CD44 and as a gatekeeper molecule whose dissociation is catalyzed by the lysosomal enzyme hyaluronidase [161]. CD44 is a transmembrane glycoprotein receptor that is overexpressed in malignant cells and promotes metastatic behavior [162]. MSN formulations used HA for gatekeeping functions with great success in preclinical models [127,128,129]. Lysozomal pronases similarly destroyed gatekeeper networks comprising polyglutamic acid, to release chemotherapeutic payloads into breast cancer cell models [136]. While exhibiting pH-responsiveness, gelatin is simultaneously broken down by matrix metalloproteinases (MMPs) in the extracellular tumor microenvironment. Such behavior is exploited to induce enzyme-stimulated gatekeeper degradation and subsequent controlled release from gelatin-coated MSNs in tumor tissue [127,134,135].

#### 3.1.3. Small Molecules

Interactions between gatekeeper molecules and small molecules within the tumor cell or microenvironment can also stimulate controlled release of encapsulated molecules. Glutathione (GSH) is one such small molecule, residing in many tumor cell types at high concentrations, relative to healthy cells [163]. GSH works in conjunction with acidic pH environments to break disulfide bonds through redox reactions [164]. Disulfide bonds can either be used to conjugate gatekeeper molecules to MSN surfaces or contribute to gatekeeper morphology. When the disulfide bond is broken through reduction by GSH, the gatekeeper molecule leaves the MSN surface or is otherwise structurally changed to open the pores and release loaded molecules. The flexibility of using and targeting disulfide bonds promoted multiple gatekeeper options with MSNs. Cyclic peptides can be used to target tumor cells while covering pore openings until undergoing GSH reduction and becoming unstructured, thus, releasing loaded molecules [164,165,166]. Proteins used as gatekeepers, such as transferrin, exhibit similar behavior in the presence of GSH and can double as targeting agents [167]. The polymer HA, when bound to MSNs through disulfide bonds, also demonstrated significant effectiveness as a tumor-specific gatekeeper [130,131]. Disulfide-bound PEG gatekeepers are similarly dissociated through interaction with GSH [168]. While gatekeeper dissociation through GSH is common, other possibilities might be employed. For example, aptamers might be used as gatekeeper molecules that selectively respond to intracellular small molecules like ATP [169].

### 3.2. External Stimuli

While internal stimuli are abundant in vivo and provide overall ease of clinical application, external stimuli were studied as a means of controlling release from MSNs. These stimuli must be provided by clinicians following administration of MSNs. Further, pharmacokinetic considerations are necessary to apply external stimuli at times when MSN accumulation in target tissue is high. Multiple gatekeeper formulations requiring external stimuli were paired with MSNs. Primarily, these gatekeepers are thermosensitive in nature, requiring an increase of site-specific in vivo temperature [170]. Induction of localized hyperthermia results in degradation or alteration in the polymer network, releasing the payload. Preclinical methods of triggering MSN gatekeeper thermosensitive release included ultrasound [137,171,172,173,174] and laser irradiation [138,175,176,177].

**Figure 4:**
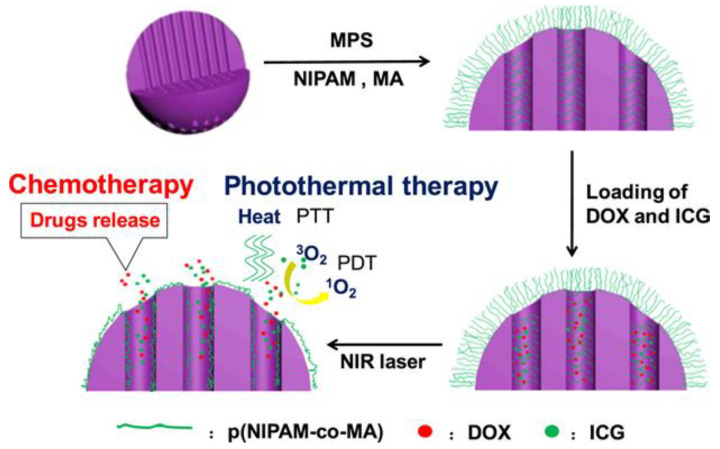
Loading and release of DOX and ICG from MSNs coated with PNIPAM copolymerized with methacrylic acid. The copolymer is abbreviated as p(NIPAM-co-MA). Copolymers are grafted from the surface of the MSN, generating dense brushes across the surface and into the porous interior. While the polymers are hydrated, off-target release is restricted. Upon heating by an externally applied NIR laser, the polymers collapse and release the loaded drug. Reprinted with permission from [143], Elsevier, 2018.

Poly(N-isopropylacrylamide) (PNIPAM), the most studied temperature-sensitive polymer evaluated for biomedical and drug delivery applications, displays a lower critical solution temperature (LCST) at 37 °C [141,142,143,144]. The polymer remains fully solvated below the LCST, but higher temperatures result in the polymer rapidly collapsing into a hypercoiled state. As the LCST is within the standard physiological temperature conditions, copolymers are often included with PNIPAM to increase its LCST to the range of 40–45 °C [142,143,144]. MSNs using grafted PNIPAM copolymers demonstrated photo-theragnostic potential with fluorescence and photoacoustic functionality. In-situ radical polymerization strategies are employed with such formulations to increase particle loading capacity and inhibit drug leakage [142]. The mechanism of release for PNIPAM is opposite of the mechanism for pH-sensitive polymers, where increased protonation swells the polymer coating to expose the MSN pores and release the loaded cargo. Rather, the native conditions maintain the polymer in its solvated state, which is then collapsed on exposure to external heating, rapidly collapsing the polymer shell and releasing the loaded drug. The mechanism for this release is proposed to result from the high density of grafting-from polymers. The dense polymer brushes can even grow short chains within the porous interior of the MSNs, swelling to block the pores in their hydrated state [143]. Upon exposure to external heating, the polymer brushes collapse, opening the pores to release the loaded drug. The thermosensitive behavior of PNIPAM can be maintained when properly copolymerized with other polymers, such as methacrylic acid, as shown in Figure 4.

## 4. Targeting of MSNs and Barriers to In Vivo Efficacy

For any NP to be clinically effective, it must reach the target site being sequestered in off-target tissue or otherwise cleared from the body. To accomplish this goal, multiple disease-site targeting strategies were implemented in preclinical nanomedicine studies. The primary options could be divided into three categories—passive targeting methods, which take advantage of innate behaviors and characteristics of diseased tissue [178]; active targeting methods, which utilize various molecular ligands that seek specific characteristics of target cells [179]; and magnetic target, which uses magnetic fields to draw susceptible NPs to disease sites [180]. While active and magnetic targeting approaches require specific design or modification of synthesized MSNs, passive targeting is assumed to be acting at all times in vivo, based on the innate physiological properties and behavior. Targeting strategies can also be combined in attempts to improve theragnostic efficacy, such as through conjugating antibodies to magnetically active NP formulations [181].

### 4.1. Passive Targeting

Diseased tissues are frequently characterized by the gaps between endothelial cells. These gaps increase endothelial permittivity to large particles, such as NPs, while having no effect on the extravasation of small molecules [182]. Subsequent residence time of NPs within the diseased tissue is theoretically increased, as compared to the small molecules based on NP size. This phenomenon, referred to as the enhanced permeability and retention (EPR) effect, remains the driving force for a significant number of NP applications, since its discovery in the 1980s [183]. Passive targeting effectiveness is primarily influenced by MSN size, shape, and surface charge [184]. While preclinical evidence supporting the efficacy of the EPR effect is vast, clinical studies showed that passively targeted NPs are not more effective than lone molecules [185,186,187,188]. The differences in efficacy in preclinical animal models and human subjects are thought to be the results of innate differences in physiology and tumor features. Irregular or lack of blood flow in solid tumor bodies along with passive targeting inability to target metastatic tumor cells are additional sources of EPR ineffectiveness [188]. While MSNs did not undergo clinical trials to assess their passive targeting effectiveness in humans, current evidence from other NP formulations suggest that active targeting is a better strategy.

### 4.2. Active Targeting

Malignant cells possess different characteristics than healthy tissue. Examples of such traits include altered metabolism, under- or overexpression of proteins and signaling molecules, or changes in gene expression. Conjugating molecules that selectively respond to these specific markers onto NP surfaces allows for active targeting of diseased cells. Attaching the MSNs to the targeting ligands can occur by multiple strategies. For example, reagents containing functional groups can be attached to MSN surfaces. These selected reagents should possess reactive functional groups that promote bond formation with the specific targeting ligand of interest ligands. For example, *N*-succinimidyl (maleimidomethyl) cyclohexanecarboxylate contains a reactive amine group that forms maleimide bonds with thiol groups, such as those found on the amino acid cysteine [38]. Functional groups on the unconjugated surface of the MSNs might be similarly used for attaching active targeting ligands [64]. Non-chemical methods, such as physisorption, also demonstrated success in conjugating active targeting ligands to some MSN formulations [189].

Employing active targeting strategies significantly enhances NP accumulation near and engulfment by target cells. Several molecules were used to actively target MSNs to specific cells. While most such options utilize specific ligand–receptor interactions, a few unique cases warrant special attention. A discussion of different active targeting molecules with apparent advantages and disadvantages is provided. For additional reference, Table 2 below presents several active targeting molecule options that were successfully used with MSNs.

#### 4.2.1. Monoclonal Antibodies

The most prevalently used proteins for active targeting in nanomedicine, as well as for NP-free cancer therapies, are monoclonal antibodies (mAbs). Separate from NP applications, mAbs are well researched and clinically used against a myriad of diseases, for decades [222,223,224]. mAbs possess antigen binding sites that are highly selective for surface proteins on target cells [225]. Such selectivity permits specific interactions between mAbs and cells presenting the surface protein of interest, while the mAbs effectively ignore cells that do not produce the target antigen. This specificity for target cells, in addition to mAbs is less likely to illicit immune responses, as these are derived from human B cell antibodies [225] and serves as the primary reason for choosing mAbs as targeting molecules for MSN theragnostics. If a new target protein is identified on the surface of a diseased cell, mAbs that specifically bind to it can be created [226]. For these benefits, there are notable drawbacks to using mAbs, mostly stemming from their large size, relative to other active targeting molecules. The average mAb is around 10 nm in size and has a molecular weight of ~150 kDa [227]. Such proportions affect NP performance in two ways—(1) increasing the diameter of the NP, which can alter its physiological fate, and (2) decreasing the number of mAbs that can bind to a single NP. The resulting lower ligand:NP ratio, relative to the ratio seen with smaller targeting ligands, can limit the targeting ability of NPs or increase the likelihood of NP sequestration by the immune system [228,229,230]. Despite these apparent difficulties, numerous MSN formulations utilizing mAb active targeting demonstrated significant tumor-specific delivery and therapeutic effect [190,191,192,193,194].

#### 4.2.2. Antibody Fragments

To overcome the size obstacle experienced with mAbs while still taking advantage of antibody targeting capabilities, antibody antigen-binding fragments (Fabs) can be used [231,232]. Fabs are the binding domains in the variable region of the antibody [233] and, thus, are the primary targeting factor of antibody action. By using Fabs as opposed to whole antibodies, not only is the size of the entire NP reduced as compared to mAb conjugations, but the ligand:NP ratio is subsequently increased as well, thus increasing the likelihood of engulfment of MSNs by target cells. MSN formulations successfully used antibody fragments as their targeting components for treating or detecting preclinical ovarian cancer [195] and breast cancer models [196], respectively.

#### 4.2.3. Peptides

Similar to proteins, peptides possess the ability to selectively interact with diseased tissue cells. The primary advantage peptides have over whole proteins is their much smaller size. The smaller size promotes a more favorable ligand:NP ratio, and subsequently, a more favorable ratio of targeting ligand to target. Smaller size does impart some disadvantages, including high hydrophobicity and a lack of secondary structures, which result in less overall stability as compared to whole proteins [234,235]. Many NPs employing peptides as their active targeting agent overcome these challenges. As seen with proteins, receptor-ligand interactions between peptides conjugated to NP surfaces and specific receptors on target cell surfaces facilitate active targeting. A commonly used receptor-targeting peptide in NP therapies is the RGD peptide, which was shown to target upregulated integrins on tumor cells [236,237]. For tumor cells, integrins enhance proliferation, adhesion, angiogenesis, invasion, migration, and inhibition of apoptosis [238]. Integrins that promote these behaviors, namely the α_v_β_3_ integrins [239,240], are upregulated, promoting distinction between tumor cells and healthy tissue cells [241]. The ability of RGD-MSN nanovehicles to selectively target these integrins and deliver loaded molecules to tumor cells is well established in numerous studies over myriad studies, including several recent advances [128,202,242,243,244,245].

Ligand–receptor targeting is not the only method through which diseased cells might be distinguished from healthy cells. Often, diseased cells will alter the microenvironment in which they reside. For example, tumor cells increase the acidity of their extracellular environment from pH 7.4 (e.g., standard physiological pH) to between pH 6.0–6.8 [146,246]. Largely, this phenomenon is due to the altered metabolism [145,247]. Similar acidification of the extracellular space is seen in inflamed tissues [248]. The lower pH around such diseased cells serves as an identifier for pH-specific interactions between administered agents and the target cells. pH-low insertion peptides (pHLIPs) undergo significant changes in their secondary structure when exposed to (a) cellular membranes and (b) a decrease in pH [249,250,251,252]. This targeting mechanism operates independent of specific receptors, thus, potentially improving patient-to-patient outcomes as compared to receptor-dependent targeting options. Through conjugating pHLIPs on the outside of NPs, researchers took advantage of the pH-specific targeting nature of these peptides [253,254]. pHLIP-conjugated silica NPs with a chitosan gatekeeper is used to treat in vitro and in vivo ovarian cancer cells through dual-action pH-dependence, as both the pHLIPs and the chitosan selectively responded to the lower pH of the tumor environment [38]. Similar MSNs were used for identifying preclinical pancreatic [62] and breast cancer models [255].

Another technique for targeting diseased cells uses cell-penetrating peptides (CPPs, a.k.a. protein transduction domains or PTDs). Commonly derived from natural sources such as viruses, CPPs can act on cells in many ways. CPP action is typically independent of receptor-specific interactions. Their mechanisms does depend on the target cell type or molecules the CPPs might be carrying, among other factors [256,257]. CPPs act on cellular membranes, as a result of the hydrophobic or electrostatic interactions between the CPPs and membrane phospholipids [208]. There are two primary ways to use CPPs in NP therapies—nonspecific CPP targeting or specific CPP targeting. The former uses CPPs that can enter into nearly any cell type, while the latter employs a CPP that is specific to the diseased target cell. When using a nonspecific CPP, the peptide must be shielded or inactivated to limit NP uptake by off-target cells. When in the extracellular microenvironment of the diseased cells, the CPPs can be unveiled or activated by extracellular markers (e.g., acidic pH or extracellular enzymes), permitting the CPPs to act on the cells and granting NP entry [258]. Both specific and nonspecific CPPs were paired with MSNs to target myriad tumor models [189,203,204,205,209], or to cross biological barriers such as the intestinal mucosal lining [206,207].

#### 4.2.4. Aptamers

MSNs might also be functionalized with aptamers, short strands of DNA or RNA that can recognize and interact with malignant cell markers [259,260,261] via an active targeting mechanism. Aptamers are highly tunable, due to their 20–60 nucleotide size [262], which increases their potential to bind to myriad targets. The binding between aptamers and target receptors occurs in a manner similar to protein–receptor interactions. Generally, aptamers target upregulated or mutant proteins found on diseased cells or in the correlating extracellular microenvironments. Examples of such proteins that are successfully targeted by aptamer-functionalized MSN formulations include nucleolin [60,210], mucin-1 [211,214], HER2 [212], and PTK7 [213].

#### 4.2.5. Small Molecules

The examples presented thus far demonstrate the targeting effectiveness of large biomolecules. While these larger molecules comprise a significant majority of applied active targeting strategies, small molecule approaches can also be used in targeting diseased cells [263]. The choice of small molecules for active targeting must match with an appropriate tumor receptor. As previously discussed, HA binds to the overexpressed CD44 receptors on tumor cells, making it an ideal choice for a small molecule targeting agent with MSNs [88,221]. It should be noted that short HA chain lengths should be used when using HA for targeting, as they increase the engulfment efficiency [264]. Tumor cell overexpression of folate receptor α encourage the use of folate as another small molecule active targeting option for MSNs [202,215,216,217,218,219,220].

### 4.3. Magnetic Targeting

Beyond physiological or molecular targeting options, magnetic manipulation of metallic agents can be used to induce NP accumulation at target sites. By applying magnetic fields around the desired site, NPs are attracted to the area, remaining in the tissue long enough for their action (such as delivery of loaded molecules) to occur. SPIONs are the primary component for magnetic targeting. While many approaches use such NPs alone or modified [180], MSNs can be used to encapsulate SPIONs or other iron-based NPs to take advantage of magnetic targeting [129,265,266]. Superparamagnetic iron oxide nanoparticles (SPIONs) are also incorporated into MSNs as a means of inducing intracellular hyperthermia, while enhancing the effect of chemotherapies [267]. There are two principle ways through which the magnetic fields can be applied to control NP accumulation—apply an external magnetic field near the target location [268,269] or implant a magnetic scaffold near the target site [270,271]. The former method is primarily for targeting areas of minimal depth, such as near the skin, while the latter permits targeting of bones or organs deep within the body of the patient. As an example, magnetic targeting for diagnostic magnetic resonance imaging (MRI), using iron-oxide-based agents was approved for off-label use by the FDA [272,273]. Trials assessing the use of lone magnetic targeting for drug delivery, to date, did not show clinical efficacy [274,275]. The largest apparent hurdle appears to be targeting deep organs (e.g., gastrointestinal or cardiovascular systems). One proposed method to increase efficacy is to use active targeting methods in conjunction with magnetic targeting. For example, MSNs containing iron NPs and functionalized with transferrin demonstrated significant ability to cross the blood–brain barrier and deliver chemotherapeutics to brain gliomas [197].

## 5. Challenges, Directions, and Conclusions

For all the advantages that MSNs possess, several challenges need to be overcome prior to clinical translation. First, more human trials are needed to test the toxicity and clinical efficacy of agents that were shown to have more therapeutic potential than the lone molecules in preclinical animal models. The nature of passive targeting by the EPR effect in humans must be better understood to ensure a greater clinical effect of NP treatments [276]. This is especially crucial for enhancing the translation of nanomedicines from preclinical models (e.g., mice) to human patients. Further research into the interplay between MSN size, shape, porosity, and surface chemistry, alongside impact on tissue penetration, cellular uptake, and release kinetics is critical for optimizing MSN formulation for greatest theragnostic effect. Additionally, assessment of gatekeeper molecule toxicity and biocompatibility in humans is necessary. While such analysis was performed for some of the described gatekeeper molecules in non-gatekeeper formulations [277,278,279,280,281], many are yet to be tested for human safety. In order for MSN formulations to undergo FDA investigational new drug (IND) pathways, reproducibility of particles on large scales is necessary. To date, MSN formulations are synthesized in small-scale batches, limiting translation to clinical environments. Understanding the regulatory synthetic requirements for production of clinic-ready MSNs is critical, as MSN technology advances.

MSNs serve as a multifunctional platform for theragnostic nanomedicine. Innately, MSNs possess high loading capacities, stable porous structures, and high surface-to-volume ratios. Together, these characteristics provide a strong foundation for MSN encapsulation of drug or dye molecules, or even other smaller NPs. By tailoring the MSN properties during and after synthesis, in vivo behavior and clinical efficacy might be optimized. Surface modification of functional groups or application of gatekeeper molecules might prevent toxic interactions between the MSN core and the host environment. Simultaneously, the gatekeeper controls the encapsulation and release of loaded molecules in a stimuli-specific manner. The ability to functionalize MSNs with various targeting options is also highly favorable for enhancing clinical outcomes. Many active targeting strategies demonstrated significant preclinical potential with MSNs, with limited off-target accumulation. Based on the current body of evidence, MSNs that are within the 20–80 nm diameter range paired with stimuli-responsive gatekeepers and active targeting moieties, might operate as the most clinically beneficial formulation. As research continues to address the apparent presented obstacles, MSN formulations will see growing prominence in clinical nanomedicine, which might correlate to enhanced clinical outcomes and patient care.

## Figures and Tables

**Table 1:** Selection of MSN Gatekeepers.

Molecule	Stimuli/Response	References
Chitosan	pH/Protonation of primary amine results in swelling from like charge repulsion	[38,61,62,119,120,121]
Polyvinyl Pyridine	pH/Protonation from acidic environment induces hydrophilic behavior	[122,123]
Poly(L-histidine)	pH/Amine group protonation results in degradation	[90,95]
Poly(acrylic acid)	pH/Protonation results in shrinking of polymer chain	[124,125,126]
Hyaluronic Acid	Enzymes and GSH/Hyaluronidase breaks down HA polymeric network or GSH breaks disulfide bond between HA and MSN	[127,128,129,130,131]
Gelatin	pH & Enzymes/Protonation of amine groups results in network swelling while MMPs degrade breakdown polymeric chains	[127,132,133,134,135]
Polyglutamic Acid	Enzymes/Degraded through catalytic interaction with pronases	[136]
Polydopamine	pH and Thermosensitivity/Acidity degrades polymer while high energy from ultrasound or laser stimulation may induce unstable state	[112,137,138,139,140]
Poly(N-isopropylacrylamide)	Thermosensitivity/Temperatures above critical temperature result in hypercoiled state of polymer, exposing MSN pores and surface	[141,142,143,144]

**Table 2:** Active Targeting Molecules Used with MSNs.

Molecule Class	Targeting Molecule	Method of Action	References
Proteins	mAbs	Specific binding with surface antigens on target cells	[190,191,192,193,194]
	Fabs	Specific binding with surface antigens on target cells	[195,196]
	Transferrin	Binds to overexpressed transferrin receptor 1	[167,197,198,199]
	Affibodies	Engineered proteins designed to selectively bind to specific receptor on target cell	[61,200]
	Heparin	Anti-angiogenesis agent and ligand-receptor targeting with overexpressed surface heparanase	[201]
Peptides	RGD	Overexpressed integrin α_V_β_3_ are selectively bound	[128,166,202]
	pHLIPs	Transmembrane insertion resulting from acidic tumor microenvironment	[38,62]
	CPPs	Specific or nonspecific interaction with the cell membrane or proteins on its surface	[189,203,204,205,206,207,208,209]
Nucleic Acids	Aptamers	Overexpressed surface receptor proteins (e.g., GLUT1) are targeted by designed nucleic acid chains	[60,210,211,212,213,214]
Small Molecules	Folate/Folic Acid	Ligand-receptor targeting between folate and folate receptor α	[202,215,216,217,218,219,220]
	Hyaluronic Acid	Overexpressed CD44 on tumor cell surfaces binds with HA	[88,128,221]

## Abbreviations

NP	Nanoparticle
MSN	Mesoporous Silica Nanoparticle
CTAB	Hexadecyltrimethylammonium Bromide
TEOS	Tetraethyl Orthosilicate
TPOS	Tetrapropyl Orthosilicate
TMOS	Tetramethyl Orthosilicate
SAM	Self-assembled Monolayer
DLS	Dynamic Light Scattering
TEM	Transmission Electron Microscopy
BET	Brunauer–Emmett–Teller
RES	Reticuloendothelial System
AR	Aspect Ratio
TEA	Triethylanolamine
PEG	Poly(ethylene glycol)
CRP	Controlled Radical Polymerization
ATRP	Atom Transfer Radical Polymerization
RAFT	Reversible Addition/Fragmentation Chain Transfer
NMP	Nitroxide-Mediated Polymerization
HA	Hyaluronic Acid
MMP	Matrix Metalloproteinase
GSH	Glutathione
PNIPAM	Poly(N-isopropylacrylamide)
LCST	Lower Critical Solution Temperature
EPR	Enhanced Permeability and Retention
mAb	Monoclonal Antibody
Fab	Antibody Fragment
pHLIP	pH-low Insertion Peptide
CPP	Cell Penetrating Peptide
SPION	Superparamagnetic Iron Oxide Nanoparticle
MRI	Magnetic Resonance Imaging
IND	Investigational New Drug
